# Barriers to and Facilitators of Implementing Programs for Return to Work (RTW) of Cancer Survivors in Four European Countries: A Qualitative Study

**DOI:** 10.1007/s10926-018-9818-2

**Published:** 2018-11-22

**Authors:** Sietske J. Tamminga, Anna M. Braspenning, Anna Haste, Linda Sharp, Monique H. W. Frings-Dresen, Angela G. E. M. de Boer

**Affiliations:** 1 Department: Coronel Institute of Occupational Health, Amsterdam Public Health Research Institute, Academic Medical Center, University of Amsterdam, Amsterdam, The Netherlands; 2 Institute of Health & Society, Faculty of Medical Sciences, Newcastle University, Newcastle upon Tyne, UK

**Keywords:** Neoplasms, Return to work, Implementation, Intervention, Focus groups

## Abstract

*Purpose* Implementation of return to work (RTW) programs for cancer survivors has proved to be challenging. The purpose of our study was to gather experiences about barriers to and facilitators of implementing RTW programs for cancer survivors in four European countries. *Methods* Separate multidisciplinary focus groups were held in Belgium (n = 8), the Netherlands (n = 8), Ireland (n = 6), and UK (n = 4) in 2017 and included among others a physician, and a representative of an employer, a cancer society, and the government. Primary focus of thematic analysis was what could be done to improve the implementation of RTW programs for cancer survivors. Analysis used the ‘Arena in work disability prevention model’ as the conceptual framework. *Results* Many barriers to and facilitators of implementing RTW programs for cancer survivors were described including the personal, workplace, healthcare and legislative system as well as the overall societal and political context. That is, for example cooperation between stakeholders, time, money and ability issues at the workplace, and insufficient/inadequate legislation. Insufficient knowledge of cancer and its implications for work was identified as an overarching theme in all countries leading to stigma, misconceptions and lack of communication. This was mentioned in relation to the workplace, personal and healthcare system, and in the overall societal context. *Conclusions* Results indicate that a prerequisite for implementing RTW programs is raising sufficient knowledge regarding cancer and its implications for work. Greater knowledge could be a first step to better implement RTW programs which may result in better supporting cancer survivors with their RTW .

## Background

Each year, an estimated 3.7 million new cases of cancer are diagnosed in Europe with approximately half of these occurring at working age [1, 2]. Due to advances in early detection and treatment the overall number of people who survive cancer is increasing [1]. However, many cancer survivors still face long-term symptoms and impairments after treatment such as fatigue, cognitive limitations, and/or physical or functional limitations [3, 4]. In this context, the cancer survivors’ quality of life (QOL) becomes a crucial issue [5–7].

One important aspect of QOL, being employed, may be hampered by symptoms and impairments with respect to cancer treatment [8]. Overall, cancer survivors have a 1.4 higher risk of becoming unemployed [9]. This is unfortunate, as return to work (RTW) is seen by cancer survivors as an important step in the transition from illness to normal life [10]. For employers and the society at large, it is economically important to encourage cancer survivors to RTW whenever possible [11].

There has been increasing interest in improving RTW outcomes for cancer survivors by means of programs [12]. By programs we mean any type of policy, initiative, or intervention (big or small) aimed at assisting cancer survivors in their RTW process or providing employers with programs which assist them in the work resumption of cancer survivors [6]. A complicating factor is that RTW involves many different stakeholders who may interact with each other to support or undermine appropriate RTW conditions (e.g. the occupational physician and healthcare professionals). RTW for cancer survivors is also embedded in the overall legislative system and societal context [13]. Therefore, to optimize the RTW of cancer survivors, developing and implementing programs requires close collaboration between different stakeholders at different levels e.g. the individual, organizational, and contextual level [14].

In light of this complexity, the implementation of individual RTW programs for cancer survivors has proved to be challenging [15, 16]. Implementation includes the process of putting programs to use or the process of integrating new programs within a setting [17–19]. Implementation can be affected by stakeholders representing different levels having different perspectives [20]. For instance, healthcare professionals may hesitate to discuss RTW during cancer treatment while the occupational healthcare professionals are addressing RTW at an early phase [21]. For a cancer survivor, the initial advice received by their own healthcare professional may negatively affect implementation of the RTW program by occupational healthcare professionals. In addition, depending on a country’s legislation and healthcare system, the degree of involvement in a RTW program of these stakeholders does differ [20]. We consider therefore that the context in which RTW programs are to be implemented might be different among European countries. Therefore, in attempting to better understand what hinders and, more importantly, may help better implement RTW programs, it is important to not limit consideration to a single country with its specific legislation and healthcare system but also include a diverse set of countries in terms of legislation and healthcare system to be able to complement findings. Additionally, to the best of our knowledge, previous research has focussed solely on the implementation success of individual RTW programs for cancer survivors [15, 16]. It would be of added value to study the barriers to and facilitators of a large and diverse set of RTW programs for cancer survivors to see whether overarching themes can be identified.

The purpose of our study was therefore to gather experiences about barriers to and facilitators of implementing RTW programs for cancer survivors in four European countries from a multi-stakeholder perspective.

## Methods

In this study, separate multidisciplinary focus group interviews were performed in Belgium, The Netherlands, Ireland, and the United Kingdom (UK). As not much is known about the implementation of RTW programs for cancer survivors [22] focus groups were chosen as they allow for an initial exploration of a topic. These countries were selected because they had examples of RTW programs for cancer survivors in place [23] and were diverse in terms of their legislation and healthcare systems [13, 24–26]. The Dutch system is characterised by employers and employees having both the responsibility to achieve a sustainable RTW, which means that both parties have rights and obligations. During the first 2 years of sick leave, an employee on sick-leave receives at least 70% of his/her income from his/her employer. In general, physicians do not pay much attention to RTW. In contrast, the Belgium system was traditionally characterised by protection of income paid by the insurance service. However, at the time of this study, new legislation was put in place to shift from protection towards action. In Ireland, an employee on sick leave can apply for statutory Illness Benefit, entitlement to which is based on the individual’s social insurance contributions. If awarded, the employee receives a payment of approximately 60% of their pre-illness income for a maximum to 2 years. Employers sometimes provide sick pay; the amount and period of provision is at the employer’s discretion. Little attention is paid to RTW after cancer by physicians, employers or the state. In the UK, for most workers, employers are obliged to pay statutory sick pay for the first 26 weeks of sickness absence. Many employers supplement this, paying greater amounts for a longer period. Physicians, employers and the state are not particularly focused on RTW after cancer. The cancer charity Macmillan provides many resources supporting RTW for patients and employers. The study was reported following the COnsolidated criteria for REporting Qualitative research (COREQ) checklist [27]. Ethical approval was not required in each individual country as the Medical Research Involving Human Subjects Act does not apply to this study, as focus groups participants were experts.

### Focus Group Participants

Experts were eligible to participate in the focus groups if they: (1) had experience in RTW policies, strategies and programs for cancer survivors; (2) were familiar with how the policies, strategies and programs for cancer survivors were operating or operated in their country; and (3) were working for Occupational Health (OH) services, research institutes focused on employment or other relevant networks/non-government organisations, or were registered as an occupational safety and health service provider, labour inspector, safety technician, healthcare provider, government representative, trade union representative, employer representative, or representative of agencies focused on employment.

All authors, the European Agency for Safety and Health at work (EU-OSHA) and the countries’ focal points of EU-OSHA nominated eligible experts. Fifty-one eligible experts were approached by email with an information letter concerning the purpose of the study and the focus group procedure as well as relevant background information about the study. A sampling approach was conducted on the experts’ background and on those experts that responded to the invitation in order to create a multidisciplinary focus group. Six invited experts did not respond, 19 could not participate on the given date/time and none responded indicated that they did not want to participate.

### Focus Group Procedure

Focus groups were held in April, May and June 2017 at a University or Cancer Society in each country. The duration of the focus group sessions was 2.5 h each. The focus group in the Netherlands was held in Dutch/Dutch, in Belgium in Flemish/Dutch, in the UK in English/English and in Ireland in Irish/English. In all four groups, before the group formally commenced, participants signed an informed consent form, anonymity was guaranteed and the procedure and purpose of the focus group was explained. In addition, before commencement of the study, no relationship with the participants was established. However, with a few participants a professional relationship existed as some participants had collaborated with one of the study authors (ST, AdB, LS) in a previous research project or knew them quite well through work.

In each focus group a topic guide was followed (Box 1), which was composed by four of the authors (ST, AdB, AB, MdF) and EU-OSHA based on the purpose of our study, the expertise of the authors (ST, AdB, AB, MdF) and the (scientific) literature. This topic guide was not pre-tested as we believed that pre-testing would not be of added value given the broad purpose of our study. However, the topic guide was used flexibly across groups. After two groups had been undertaken we reflected on whether any changes were required to the guide before use in the later groups. It appeared that none were necessary. The group facilitator explained that “programs” meant any type of policy, initiative, or intervention big or small aimed at assisting cancer survivors in their RTW process or providing employers with programs, which assist them in the work resumption of cancer survivors.

In the Netherlands and in Belgium, one female experienced discussion leader (ST) guided the focus groups. In the Netherlands and Belgium, AB and AdB, respectively a junior and senior researcher in the field of cancer and work observed the focus groups and made notes to ease the transcription process. In the UK and in Ireland, two female experienced discussion leaders (AH, LS) guided the focus groups using the same topic guide as used in the Dutch and Belgium focus groups. Two of the three discussion leaders (ST, LS) were senior researchers in the field of cancer and work while one of them (AH) was a senior researcher in the field of cancer. All researchers present at a focus group did not represent any of the organisations nor professions present.

Box 1: Topic Guide of the Focus Groups 1. What are your experiences with RTW programs for cancer survivors?

2. What works well?

3. What are success factors of these programs?

4. What could be done to improve the programs on the level of

(a) Policy

(b) Legislation

(c) Occupational health services

(d) Healthcare

(e) Workplace

(f) Cooperation between stakeholders?

5. What could be done to improve the implementation of the programs on the level of

(a) Policy

(b) Legislation

(c) Occupational health services

(d) Healthcare

(e) Workplace

(f) Cooperation between stakeholders?

### Analysis

The interviews were digitally recorded and audiotapes transcribed verbatim. ST and AB conducted independent qualitative thematic analysis [28] using the MAXQDA qualitative analysis software package [29]. The primary focus of the thematic analysis was what could be done to improve the implementation of RTW programs for cancer survivors. At first, open codes were assigned to the focus group data that answered the research question by adding labels that represent the text as closely as possible. Then these open codes were subdivided into barriers and facilitators and into the five different “systems” according to the ‘Arena in work disability prevention model’ [30]: (1) overall societal context/culture and politics; (2) workplace system; (3) legislative and insurance system; (4) personal system; and (5) healthcare system. Constant comparison between data and coding and frequent meetings between authors (ST, AB, AdB) led to final coding and the identification of overarching themes mentioned in all systems. Final decisions were made by consensus. After classification of the coding tree and the identification of one overarching theme, all authors checked the findings and approved the final description. In the result section, findings within each theme are organised according to the five systems.

Data saturation was not explicitly sought because the objective of our study was to gather initial experiences and opinions about barriers and facilitators to implementation of RTW programs for cancer survivors in various European countries.

## Results

### Participants

The 26 participants sharing their experiences in one of the four focus groups had diverse expertise (Table 1). The groups included a vocational rehabilitation expert, (occupational health) nurse/physician, employee of a reintegration bureau specialised in RTW of cancer survivors, a researcher, an employer representative, a trade union representative, a government representative, a fund of occupational diseases representative, a worker representative, an OH representative, and included an employee of a cancer survivor society.

**Table 1:** Characteristics of focus group participants (N = 26)

Number	Country	Gender	Profession
1	Belgium	F	Researcher of occupational and environmental medicine
2	Belgium	F	Employee of a reintegration bureau specialised in RTW of cancer survivors
3	Belgium	M	Government representative
4	Belgium	M	Researcher of RTW after cancer project
5	Belgium	M	Representative of fund for occupational diseases
6	Belgium	M	Employer of a healthcare organization
7	Belgium	F	Employer of a bank
8	Belgium	M	Trade Union representative
1	The Netherlands	M	Employer of a manufacturing company
2	The Netherlands	F	Employee of a reintegration bureau specialised in RTW of cancer survivors
3	The Netherlands	F	Employee of a reintegration bureau specialised in RTW of cancer survivors
4	The Netherlands	F	Government representative
5	The Netherlands	F	Workers’ representative
6	The Netherlands	M	Occupational health physician experienced in oncology
7	The Netherlands	F	Nurse specialized in RTW and cancer
8	The Netherlands	F	Representative of an OSH research institute
1	Ireland	M	National HR division, specialist registration in occupational medicine
2	Ireland	M	Trade union representative
3	Ireland	M	Trade union representative
4	Ireland	M	Representative of cancer support organisation
5	Ireland	M	Representative of cancer support organisation
6	Ireland	M	Occupational health nurse
1	UK	F	Researcher
2	UK	F	Vocational rehabilitation expert
4	UK	F	Employee of a cancer survivor society

*F* female, *M* male

### Barriers to and Facilitators of Implementing RTW Programs

Many barriers to and facilitators of implementing RTW programs for cancer survivors were mentioned in all focus groups including all systems of the ‘Arena in work disability prevention model’ [30] (Table 2). In all but the healthcare system, both barriers to and facilitators of RTW programs were mentioned.

**Table 2:** Barriers to and facilitators of implementing RTW programs for cancer survivors

Domain	Barriers to and facilitators of implementing RTW programs for cancer survivors	IE	UK	BE	NL
Overall societal context/culture and politics	Hard to implement knowledge (dissemination/implementation)	−			
	Unclear who takes responsibility	−	−		
	Avoiding conversation	−			
	Limited/sufficient knowledge about work and cancer by employer	−/+	−	−	−/+
	Stigma		−		−
	Limited and wrong knowledge about legal right for employees				−
	Culture makes it difficult/easy to disclose				−/+
	Individually approach	−			
	Correct knowledge about possible support for employees	+			
	Culture: view about work/rehabilitation	+			
	Cooperation between stakeholders	+			
Workplace system	Level of information (too much/up to date)				+
	Avoiding conversation by employer	−	−		−
	Limited/sufficient knowledge and wrong/correct pre-assumptions about work and cancer by employer	−	−	−/+	−/+
	Stigma	−		−	
	Sufficient and correct knowledge about legal rights for employees	+			
	Organisation culture makes it easy to disclose		+		
	Individually approach	−			
	Limited/sufficient knowledge and wrong/correct knowledge about possible support for employees	−		+	+
	Time, money and ability are issues	−	−	−	+
	No OHS in SME’s	−	−	−	
	Organisation culture: view employer about rehabilitation/adjustments	−/+	−	−/+	−/+
	Role occupational physician		+	−	−
	Financial benefits/incentives				+
	Structured approach	+	+		+
	Involvement stakeholders	+	+		
	Training personnel			+	+
Personal system/personal coping	Organisation culture makes it difficult to disclose	−	−		
	Motivation, awareness and knowledge of cancer survivor	+			+
Healthcare system	Unclear who takes responsibility			−	
	Limited knowledge about work and cancer	−	−		−
	Limited/wrong knowledge about possible occupational support by healthcare providers	−		−	−
	Communication between stakeholders		−	−	
Legislative and insurance system	Level of information (too much/up to date)			−	
	Financial benefits/incentives	−	+	−/+	−
	Legislation is missing	−			−

The facilitators mentioned during the focus groups are marked with a plus sign (+), the barriers with a negative sign (−)

Countries are represented with the abbreviations IE (Ireland), UK (United Kingdom), BE (Belgium), NL (the Netherlands)

#### Overall Societal Context/Culture and Politics

In all four countries limited knowledge about cancer and its impact on work was mentioned as a barrier to implementing RTW programs but not to the same extent. In the Irish focus group the participants were rather optimistic about this point and thought that the real challenge is that information, much as a book, needs updating. In contrast to other countries, where it was felt that society at large is insufficiently aware of what cancer survivors can and cannot do, largely holding the opinion that an individual cannot work for months when diagnosed with cancer:

We also have people who get cancer, who immediately go on sick leave (….). While we know when you remain working, maybe in the beginning or later, it can even help you with the recovery, but they will not do that (..) so I think when we talk about providing information, it means also that employees will be provided with information about what they can do even if you get that diagnosis. [The Netherlands, #7]

In addition, the society at large is unsatisfactorily aware of differences between cancer survivors as a participant in the Belgium focus group pointed out:

But it is very difficult, THE cancer patient does not exist. There are so many different ones in addition to psychological ways of experiencing that episode [Belgium, #1]

However, according to participants it is difficult to implement such knowledge. Insufficient knowledge in combination with stigma can lead to negative assumptions being made by people in the overall society, people not wanting to disclose that he or she is a cancer survivor, and people in the overall society avoiding conversations about cancer and RTW. Limited knowledge also leads to fear of taking responsibility as a participant in the Irish focus group explained:

And that’s where I think you see the fear. Neither side [employer/employee] wants to be the first one to initiate it [the conversation], because they’re afraid that they might do something wrong, or say something wrong [Ireland, M3].

In the UK focus group the difficulty with taking responsibility was referred to as a lack of clarity regarding who/which organisation would be responsible for meeting the costs of resources to help the employee diagnosed with cancer back to work. In the Dutch focus group, insufficient knowledge was considered to lead to the assumption that the government would take care of cancer survivors, not realising that the welfare state is cutting back and that cancer survivors might have obligations to the employer/social security system to make an effort to RTW as well.

On the positive side, facilitators when implementing RTW programs were described as (i) getting people to believe that it is possible to work during/after cancer treatment and (ii) understanding of the benefit to society at large that cancer survivors continue working when possible. Additionally, understanding what the individual cancer survivor requires to stay employed was believed to facilitate the implementation of RTW programs. Furthermore, creating cooperation between all the different stakeholders involved was also believed to facilitate the implementation of RTW programs. That is, optimizing the interaction between the different systems of the overall societal context.

#### Workplace System

Insufficient knowledge about work and cancer was mentioned as a barrier to implementing RTW programs in the workplace system as well. As mentioned above, this led to the same negative assumptions being made as to what a cancer survivor can and cannot do. Avoiding the conversation about RTW was mentioned specifically at workplace level in relation to the employer, HR-manager and colleagues. Insufficient knowledge about cancer and its impact on work furthermore entails not having enough understanding by the employer and/or their representative of rights and responsibilities and for the need to support employees diagnosed with cancer to stay at work.

In most countries the fact that Small and Medium Enterprises (SME’s) had no Occupational Health and Safety (OHS) services in place and that SME’s may have less flexibility to accommodate work changes in work for cancer survivors was mentioned as a barrier to implementing RTW programs. Furthermore, in large companies with OHS services in place it was believed that change or reduction in the role of occupational physicians by employers has impacted on their ability to influence RTW of cancer survivors, as was discussed by a Dutch participant:

Because I think the occupational physician is really a key figure with respect to return to work, staying at work, but that role has changed, so much. Stripped down.

[The Netherlands, #5].

Mentioned as facilitators to implementing RTW programs were: (1) employers who have a clear understanding of legal rights and obligations; (2) employers who have a supportive culture making it easier for survivors to disclose; and (3) employers staying up-to-date about possibilities to RTW after cancer, as was pointed out by a Irish participant: Mentioned as facilitators to implementing RTW programs were: (1) employers who have a clear understanding of legal rights and obligations; (2) employers who have a supportive culture making it easier for survivors to disclose; and (3) employers staying up-to-date about possibilities to RTW after cancer, as was pointed out by a Irish participant:

Yes, there’s comfort in knowing that it’s [policy] there, if or should you need it, but you have to know that it’s there, should you need it in the first place. And I think that’s the challenge. [Ireland, M1]

Furthermore, having a structured approach securely in place before an employee is diagnosed with cancer but which could be tailored to the individual, and in which all relevant stakeholders are involved and properly trained, was mentioned as a facilitator. This is however, challenged by time, money, and ability issues. Additionally, it is challenged by an organisation that has a negative view on rehabilitation and adjustments as was explained by a Dutch participant:

And what I see during dismissals is that they are really looking for cherry picking who they will keep and who not. That is also, I see a lot of people [who were diagnosed with cancer] of 50+ being made redundant. [The Netherlands, #7]

#### Personal System

In the personal system, the cancer type and/or an organisational culture might contribute to deciding not to disclose that you have cancer or difficult to disclose what you need as a cancer survivor. These are perceived as barriers to implement programs specially for cancer survivors, as was explained by one of the participants:

I think it depends on the cancer. If you have a brain tumour, you might not want to tell your employer, especially depending on what you do. [UK, #2]

On the contrary, the motivation, awareness, and knowledge of a cancer survivor were mentioned as facilitators for implementing RTW programs, as was explained by one of the Irish focus group participants:

But I suppose that personal motivation would have a very powerful impact on them [the employer], to facilitate somebody coming back to work. [Ireland, F3]

#### Healthcare System

The participants mentioned only barriers to implementing RTW programs in the healthcare system. Insufficient knowledge about work and cancer was mentioned as a barrier leading to lack of clarity who takes responsibility and which healthcare professional should start the discussion on RTW. In addition, uncertainty of healthcare professionals about whether and what RTW advice to give to cancer survivors, insufficient time to have a conversation about RTW, incorrect or insufficient knowledge about negative consequences for the work situation of cancer survivors of their advice given, were also mentioned as barriers as was pointed out by a UK participant:

Sometimes you hear that cancer survivors say that they’ve been discouraged from going back to work by their clinicians. That happens very often, even if it might be in the patient’s interests. Clinicians will just look at cancer as a condition, and they don’t see any other aspect. [UK, #2].

According to the participants this often led to healthcare professionals being overprotective and not supporting RTW as its negative impact is not seen by healthcare professionals. Furthermore, the lack of communication either between different healthcare professionals or between the healthcare professional, occupational physician and employer, was mentioned as a barrier as the stakeholders often do not have the same goal, are not aware of what is happening and do not have the same information.

It is essential that there is communication, a possibility of easy communication between the three involved physicians, to be honest. Between the treating physician, the insurance physician and the occupational physician. There is still no systematic solution for this.

(Belgium, #4)

#### Legislative and Insurance System

Various barriers and facilitators of the legislative system or absence of legislation of each country were discussed in great detail. In the Belgium group the discussion centred around the fact that employers could fire sick-listed employees, that it was too easy for employers to put no effort in RTW and that legalisation was so complicated that nobody understood it. The employer having no responsibility in relation to survivors RTW (e.g., no sick pay regulation) was also mentioned in the Irish focus group. In contrast, in the Dutch focus group, the obligation of the employer to pay two years of salary in case an employee is sick-listed was discussed as a barrier as employers are reluctant to hire employees diagnosed with cancer because employers fear having to pay salary if someone has long-term side effects of cancer treatment and/or a recurrence. In addition, the self-employed not being obligated to be insured and thereby not having access to OHS and thus no infrastructure in which an RTW program could be implemented was identified as a barrier. In the UK focus group, a driver for the employer to support RTW was mentioned to avoid their insurance premium rising, as illustrated by the following quote:

I was in a very privileged position of working with companies who had paid for insurance [to provide sick pay for employees absent from work due to illness], so this was a service that we delivered under the umbrella of the employer’s own insurance. Their employees have a financial safety net. The way that we focussed it was, “Yes, you can ignore helping this person get back to work”. The ‘but’ was that, just as, if you’re in lots of car accidents, your premium next year will go up, there’s a financial piece underlying it for the employer if they can get a person productively back to work. [UK, #1]

In both the Belgium and Dutch groups, privacy legislation was considered to hamper open communication and was therefore seen as barrier, as was illustrated by the following quote:

..because the existing legislation looks like most of the time to be focusing on the protection of the employee but actually it is resulting in barriers regarding return to work, and in addition the legislation is not always in line with the needs of the employee or employer. [Belgium, #9]

Finally, in the Irish, Belgium and Dutch focus group difficulties working part time and receiving disability benefits was discussed as a barrier to implement RTW programs as was explained by an Irish participant:

if you’re not coming back on a full-time basis, you’re coming back on a part-time basis, you’re actually losing an element of money, but you can only get social welfare if you work a certain amount of hours per week.[Ireland, M3]

## Discussion

The purpose of our study was to gather experiences about barriers to and facilitators of implementing RTW programs for cancer survivors in four European countries. Many barriers to and facilitators of implementing RTW programs for cancer survivors were mentioned, which could be mapped out to all the different systems of the ‘Arena in work disability prevention model’ that is the personal, workplace, healthcare and legislative system as well as the overall societal and political context. For example, cooperation between stakeholders, time, money and ability issues at the workplace, and insufficient legislation. Insufficient knowledge of cancer and its impact on work was identified as an overarching theme in all countries, which was mentioned in the workplace, personal and healthcare system as well as in the overall societal context.

### Strengths and Limitations

A strength of this study comprises the constitution of the focus groups, which include all relevant stakeholders. It furthermore comprises the stakeholders’ up-to-date experiences with the implementation of RTW programs for cancer survivors as well as their knowledge of the national legislation system and health care system. Additionally, we consider using the ‘Arena in work disability prevention model’ as a conceptual framework as a strength as it allows to map out all barriers to and facilitators of implementing programs in all systems involved in a systematically way. Finally, another strength of this study is the inclusion of stakeholders from various European countries [30]. This enabled us to gather an initial idea about commonalities and differences in barriers to and facilitators of implementing RTW programs of cancer survivors across countries that differ to a great extent in terms of legislation and health care system. As a limitation of our study, we did not strive for data saturation. This means that our results could not be generalised to the situation in each country. As we included four countries only, it could not be generalised to other (European) countries. However, our results could be used as a starting point to improve the implementation of RTW programs for cancer survivors. Additionally, it could be used to further identify barriers to and facilitators of the implementation of RTW programs on a larger scale and more systematic way. This future research could provide us with a complete and generalizable picture on how to improve the implementation of RTW programs for cancer survivors. Additionally, there was a pre-existing professional relationship between the focus group leaders and some of the participations. This was inevitable as we thought it very important that an experienced researcher in the field of cancer and work would lead the focus groups. We think it is unlikely that this professional relationship could have influenced the findings as the relationship was of equal value. This is furthermore supported by the fact that in general, group participants including those with a professional relationship with the group leaders, were open, frank and willing to share views and experiences.

### Interpretation of Findings

First and foremost, we found that in the workplace, personal, and healthcare system and in the overall societal context, insufficient knowledge about work and cancer was mentioned as barrier to implementing RTW programs for cancer survivors in all countries. This led to stigmatization of cancer survivors. In the four-phase model of implementation of the Consolidated Framework for Implementation Research (CFIR) [19, 31] knowledge and skills is mentioned in the first explorative phase and in the second adaptation and preparation phase. The first phase is characterised by developing awareness of a problem needing improvement and the second phase is aimed at adopting the proposed program. This means that to achieve successful implementation of RTW programs for cancer survivors, first awareness in each system needs to be raised and improved before the next implementation phase can be undertaken. This could, for instance, be achieved in the workplace system by means of management commitment and training/coaching activities for supervisors [19, 32], and in the healthcare and workplace systems simultaneously by means of interactive and multidisciplinary training sessions for healthcare professionals (physicians, specialised nurses, and occupational physicals) to create mutual understanding of each other’s discipline and perspective [33]. Additionally, in the societal system this could be achieved by means of providing information by the national government or the national cancer society or leading cancer charities about the possibilities to work after cancer via a national multimedia campaign, which has been done successfully by the UK charity organisation MacMillan [34].

Although a country’s legislation and healthcare system play a major role in the degree of involvement of stakeholders in the delivery of RTW programs and play a major role in which behaviour is incentivised [20], it is striking that many barriers to and facilitators of implementation of RTW programs identified by stakeholders are similar in all countries. This implies that experiences with implementing RTW programs in one country may be informative for implementing RTW programs in another country as long as the differences in legislation are well understood and taken into account. A further important observation from the study is that when considering legislation, there is no such thing as the perfect legislation. For example, the Dutch system for instance has a major (financial) obligation for employers when employees are sick-listed that leads to reluctance by employers to hire employees who are chronically ill [13, 24, 25]. However, our results indicate that when there is no legislation, it is more difficult to implement programs to support the RTW of cancer survivors as the infrastructure is not there and nobody takes responsibility. That is for instance the case in the Netherlands, UK, and Ireland for self-employed, and in all countries for SME’s. Furthermore, our results indicate uncertainty in Belgium, of who takes responsibility within the healthcare system and in the UK who/which organisation would be responsible for meeting the cost of resources to help the employee diagnosed with cancer back to work. Therefore, it seems that legislation is a pre-requisite for implementing programs that support employees with cancer with their RTW.

### Implications for Research and Practice

Derived from the interpretation of our results our most prominent recommendation for further research is to create awareness that a program in itself is not a guarantee that it is actually used in daily practice [19] and that legislation seems to be a pre-requisite for implementation of programs. In line with the key message by Main et al. [19], in order to get a program implemented it needs careful planning and a multifaceted strategy each targeting relevant stakeholders in each specific way [35] especially in the early two phases as lack of knowledge was mentioned in most systems.

In conclusion, many barriers to and facilitators of implementing RTW programs for cancer survivors were mentioned in the four focus groups. Our results indicate that prerequisite for implementing RTW programs is creating sufficient knowledge regarding cancer and work. This knowledge could be used as a first step to better implement RTW programs within each country, which may result in better supporting cancer survivors with their RTW.
